# Development and validation of a Systemic Sclerosis Health Literacy Scale

**DOI:** 10.3389/fpubh.2023.1038019

**Published:** 2023-02-23

**Authors:** Meng Zhuang, Cheng-Cheng Li, Shan-Yu Chen, Xin-Hua Tu, Lian Liu, Xi-Lai Chen, Cheng-Wei Xu, Jing Wang

**Affiliations:** ^1^Department of Epidemiology and Biostatistics, School of Public Health, Anhui Medical University, Hefei, China; ^2^Medical Data Processing Center, Anhui Medical University, Hefei, China; ^3^Department of Rheumatology and Immunology, The First Affiliated Hospital of Anhui Medical University, Hefei, China; ^4^Department of Statistics, College of Statistics and Applied Mathematics, Anhui University of Finance and Economics, Bengbu, China

**Keywords:** systemic sclerosis, health literacy, scale, chronic disease, populations

## Abstract

**Background and aim:**

Health literacy levels are strongly associated with clinical outcomes and quality of life in patients with chronic diseases, and patients with limited health literacy often require more medical care and achieve poorer clinical outcomes. Among the large number of studies on health literacy, few studies have focused on the health literacy of people with systemic sclerosis (SSc), and there is no specific tool to measure health literacy in this group. Therefore, this study plans to develop a health literacy scale for patients with SSc.

**Methods:**

This study included 428 SSc patients from the outpatient and inpatient departments of the Department of Rheumatology and Immunology, the first affiliated Hospital of Anhui Medical University and the first affiliated Hospital of University of Science and Technology of China. The formulation of the scale was completed by forming the concept of health literacy of SSc patients, establishing the item pool, screening items, and evaluating reliability and validity. Classical measurement theory was used to screen items, factor analysis was used to explore the construct validity of the scale, and Cronbach's alpha coefficient was used to assess the internal consistency.

**Results:**

Our study population was predominantly middle-aged women, with a male to female ratio of 1:5.7 and a mean age of 51.57 ± 10.99. A SSc Health Literacy scale with 6 dimensions and 30 items was developed. The six dimensions are clinic ability, judgment/evaluation information ability, access to information ability, social support, treatment compliance and application information ability. The Cronbach's alpha coefficient of the scale is 0.960, retest reliability is 0.898, split-half reliability is 0.953, content validity is 0.983, which has good reliability and validity.

**Conclusion:**

The Systemic Sclerosis Health Literacy Scale may become a valid tool to evaluate the health literacy level of patients with SSc.

## 1. Introduction

SSc is an autoimmune disease that often presents with abnormal expression of the immune system, microvascular involvement, and fibrosis of skin and visceral cells (1, 2). Patients with SSc mainly show symptoms such as hard and tight skin, swollen and painful joints, and joint dysfunction (3). Interstitial lung disease and pulmonary arterial hypertension are common complications of systemic sclerosis, and are the leading cause of death in patients (4, 5). SSc is a chronic non-communicable disease with complex etiology, insidious onset, long course and persistent disease (6). In recent years, many scholars have devoted themselves to the study of the causes and pathogenesis of SSc (7, 8). However, the causes and processes of SSc development are not fully understood, and pharmacotherapy is the main treatment, although some drugs have been shown to improve the fibrosis or complications of SSc, but not to achieve a cure (9, 10).

The increasing focus on patient-centered treatment options and patient self-care skills in the treatment of chronic diseases (11), and the requirement for patients to be able to make clear medical decisions, has made health literacy highly relevant in healthcare settings (12). Patients with limited health literacy are often accompanied by poor health outcomes, poor adherence to treatment, and underutilization of health care resources (13, 14). Many studies on health literacy and the health outcomes of chronic diseases have emerged (15–18). Multiple studies have indicated that health literacy is strongly associated with health outcomes, and that low health literacy affects an individual's ability to read and access health information (19), communicate with doctors (20), adopt a healthy lifestyle, and respond to disease warnings (21). It is observed that health literacy is a potential factor affecting the quality of life and disease management of chronic patients. In a study on chronic disease prevention in China, health literacy was linked to a reduction in the likelihood of comorbidity (22).

What is health literacy? Different scholars have developed inconsistent definitions of health literacy, and the most widely used is the definition developed by the US. National Library (23), “the ability of an individual to access, understand, and process basic health information or services to make appropriate health decisions.” In addition, WHO defines health literacy as “the ability to obtain, understand, evaluate and apply health information to make judgments and decisions in health care, disease prevention and health promotion, thereby improving the quality of life” (24). In recent years, Healthy People 2030 defines health literacy in terms of individuals and organizations, retaining the connotation of individuals finding, understanding, and using health information and services, and emphasizing the roles and responsibilities of organizations in health information and services (25).

Current research on health literacy levels in patients with rheumatic diseases has focused on systemic lupus erythematosus and rheumatoid arthritis (26, 27). The findings show an association between patient health literacy and disease activity, medication adherence, functional status, and additional health outcomes (28–30). We found only one study related to SSc patient health literacy, which, unlike traditional health literacy studies, was an assessment of e-health literacy, focusing on assessing patients' use of e-health resources and need for web-based support (31). In this study, we focused on the ability that SSc patients have to be able to make health decisions, rather than the ability to access the Web, for which no relevant literature has been found.

As we know, the awareness rate of SSc is low, and people often report being unfamiliar with the disease and need to be aware of it if they are diagnosed. SSc occurs mostly in middle-aged and old women, who generally have a low level of health awareness, lack of understanding of the disease, difficulty in correctly recognizing disease characteristics, insufficient self-management ability, low treatment compliance, and poor clinical outcomes. In addition, patients with limited health literacy use more outpatient services and are hospitalized more frequently, increasing the socioeconomic burden and additional financial burden of care (32, 33). Leonardo Martin Calderon et al. reviewed published articles on the economic impact and healthcare resource utilization associated with SSc, noting that the total annual cost of SSc ranges from $14,959 to $23,268 in the United States, which is a significant economic burden on patients and health resources (34). Therefore, we believe it is necessary to pay attention to the health literacy level of this group of SSc, raise patients' awareness, and maximize the use of limited resources as much as possible, thus improving patients' awareness of the disease, reducing the waste of medical resources, and alleviating patients' economic burden.

Health literacy scales are currently the primary measurement tool for measuring the health literacy level of study participants. Commonly used health literacy scales include “Test of Functional Health Literacy in Adults, TOFHLA” (35), “Rapid Estimate of Adult Literacy in Medicine, REALM” (36), “Brief Health Literacy Screen, SILS” (37), and “Health Literacy Scale-Europe, HLS-EU-Q (38).” Of these, TOFHLA and REALM were the first to be developed, but they mainly measured test takers' reading comprehension or numerical ability, and the tests were poorly practical and were gradually being replaced. With only three short questions, SILS takes very little time and is often used for rapid screening of clinical patients. However, the three questions included in the scale only assess the patient's understanding of medical information and do not broadly assess the patient's ability to understand and evaluate information about the disease and communicate with physicians in all areas. The HLS-EU-Q scale is aimed at the general healthy population and focuses on health education, disease prevention and health promotion for the test subjects from a public health perspective. Therefore, in order to accurately measure the wide range of competencies that SSc patients should have in the process of disease treatment, this study attempted to develop a specific health literacy scale for SSc patients from the perspective of clinical treatment, assessing patients' awareness of disease, ability to communicate with physicians, ability to obtain, understand, judge and apply medical information, and including treatment adherence and available social support.

The purpose of this study was to develop a health literacy scale for SSc patients and assess its reliability and validity, which can more widely and comprehensively evaluate the ability of SSc patients to manage their health, especially the various skills required in the course of disease treatment, and can more objectively reflect the health literacy level of SSc patients. It is hoped that this scale can provide reference for more relevant studies on health literacy of patients with SSc.

## 2. Materials and methods

### 2.1. Ethics

This study is in accordance with the Declaration of Helsinki, and the work design was approved by the Biomedical Ethics Committee of Anhui Medical University (number 20210649). All subjects agreed to participate in this study and signed the informed consent form.

### 2.2. Scale development procedure

**Figure 1:**
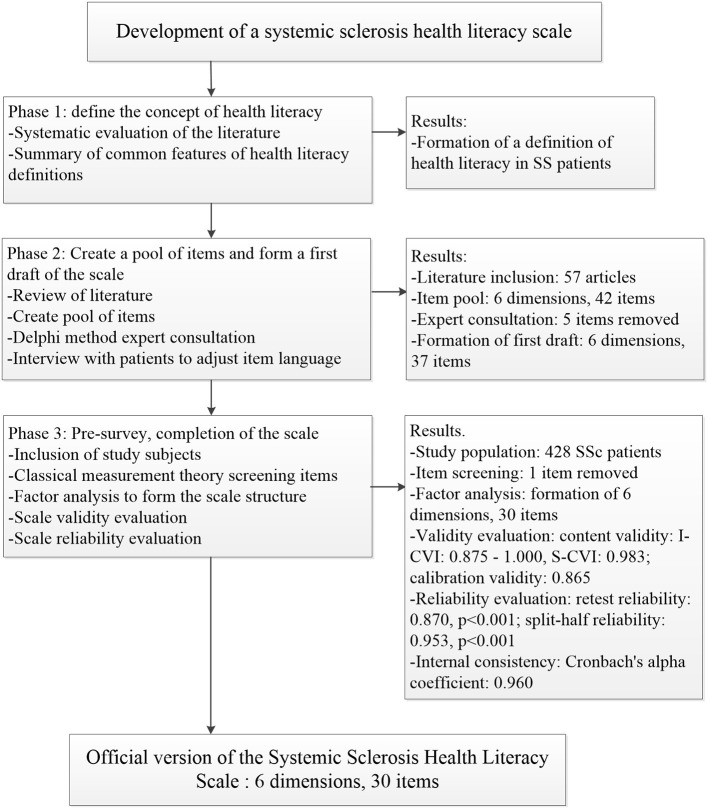
Flow chart of systematic sclerosis Health Literacy Scale development.

The study was divided into three stages: the first stage summarized published definitions of health literacy and identified the concept of health literacy in the SSc population. In the second stage, the item pool of the scale was formed based on a review of the literature; the Delphi method was used to determine the first draft items of the scale after two rounds of expert consultation; face-to-face interviews with patients were conducted to adjust the language of the scale items to form the first draft of the scale. In the third stage, main surveys were conducted to adjust the content and structure of the scale by combining classical measurement theory and factor analysis, and to evaluate the validity and reliability of the scale to finalize the development of the scale (Figure 1).

#### 2.2.1. Stage 1: Define the concept of health literacy

Previous studies have counted more than 250 definitions of health literacy, and summed up six definitions commonly used in the literature (39). The impact of individual capacity on health literacy, especially the ability to acquire and understand information, is consistently highlighted in these definitions. We summarized the common features of the different meanings of health literacy, while considering the influence of social support on health literacy, and defined the health literacy of SSc patients as: The ability of people with systemic sclerosis to access, understand, communicate, evaluate, and apply medical information or health information, including the social support available to make judgments and decisions about health care, disease management, to maintain or slow disease progression and improve quality of life.

#### 2.2.2. Stage 2: Create a pool of items and form a first draft of the scale

Literature review: From its establishment to December 2020, relevant articles about the health literacy scale were searched in Pubmed, web of sciences, China knowledge Network and Health Literacy Tool Shed. With the combination of “health literacy” and “scale,” “measure,” “assessment,” “screening” or “instrument” as the key words, 1,341 articles of Pubmed, 2,873 articles of web of sciences, 895 articles of China knowledge Network and 216 articles of Health Literacy Tool Shed were searched, mainly including the original research. Focus on the definition, dimensions and fields of the scale, remove the repetitive literature, and finally include 57 articles.

Establish item pool: Review the included health literacy scale and measurement items, divide the items according to the dimensions of access, understanding, communication, evaluation, application and social support, and delete duplicate items. Conduct expert interviews and focus groups to brainstorm, evaluate the included items, and add new items according to the previous status survey, and finally form the item pool of the scale. The item pool contains six dimensions with 42 items, namely (1) access to health information (7 items); (2) understanding health information (8 items); (3) communicating health information (8 items); (4) assessing health information (8 items); (5) applying health information (6 items); and (6) social support (5 items).

Delphi method: The Delphi method is a “back-to-back” survey in which experts evaluate the importance and applicability of items and dimensions. The authority of an expert can be calculated based on the experts' familiarity with each item and the basis of their judgment. The higher the authority of the expert, the higher the accuracy of the prediction. The degree of coordination of experts' opinions refers to whether there is a large disagreement between experts' evaluations of each item, and is commonly judged by the *p*-value of the Kendall W coordination coefficient test, with *p* < 0.05 indicating a good degree of coordination among the indicators (40). Expert selection criteria: ➀ intermediate or above professional title; ➁ bachelor degree or above; ➂ 10 years or more working experience in related professional field; ➃ willing to participate in this study and give some expert advice and guidance. In order to ensure the authority of expert opinions, 15–20 experts are planned to be invited.

Expert consultation*:* We eventually invited 16 experts, all with master's degree or above, 2 intermediate titles and 14 senior titles. The average working years of experts is 17.81 ± 5.12, and they are familiar with systemic sclerosis and health literacy. Based on the results of experts' familiarity and judgment basis for each item in the first round of expert consultation, the index judgment coefficient, familiarity coefficient and authority coefficient of experts are 0.89, 0.73, and 0.81, respectively. An authority coefficient >0.70 is an acceptable value, representing a high degree of authority of the chosen consulting expert. In this round, we have deleted four items according to expert opinions: “you can fill in the written information during diagnosis and treatment”; “you can exchange credible health information with others”; “you can judge whether the health information obtained can solve related problems”; “you can judge whether the health information said by relatives and friends is correct.” The contents of the first three items are cross-duplicated with other items, and the last one is not relevant.

After the second round of expert consultation, the expert authority coefficient is 0.85 and the Kendall W coordination coefficient of expert opinion was 0.127 (*p* < 0.001). The average importance score of each item by experts is 3.44–4.31, and the coefficient of variation is 0.105–0.280. Only the item “you can judge which daily behaviors are related to your health” had an average importance score of < 3.5 and a coefficient of variation >0.25, so it was deleted. The final scale content contains six dimensions with 37 items.

#### 2.2.3. Stage 3: Main survey, completion of the scale

Participants and sample size: The study population was obtained from the outpatient and inpatient departments of the Department of Rheumatology and Immunology, the First Affiliated Hospital of Anhui Medical University and The First Affiliated Hospital of University of Science and Technology of China, and met the diagnostic criteria for SSc established by the American College of Rheumatology (ACR) and the European League for Rheumatology (EULAR) in 2013 (41). Based on the factorial analysis requiring a sample size of 5–10 times the number of items (42), we planned to include at least 200 study subjects in each of the exploratory factor analysis and confirmatory factor analysis.

Data collection: In this study, data were collected using a convenience sampling method by face-to-face interaction with patients in the outpatient and inpatient departments of the two hospitals mentioned above from March 2021 to June 2022, using verbal questioning. In addition, a small number of patients were unable to come to the hospitals due to the epidemic, and data collection from patients was conducted using telephone questioning. During this process, the researcher used uniform language expressions whenever possible to minimize information bias.

### 2.3. Statistical analysis

SPSS 23.0 was used for correlation analysis, exploratory factor analysis and reliability evaluation, and Amos Graphics 26.0 for confirmatory factor analysis. If the continuous variable accords with the normal distribution, it is expressed by mean and standard deviation, otherwise it is expressed by median and quartile. In correlation analysis, if the variables conform to normal distribution, Pearson correlation analysis is used, and vice versa with Spearman correlation analysis.

#### 2.3.1. Item selection

Items were screened according to classical measurement theory. Classical measurement theory includes eight methods, and items that meet five or more of these retention criteria will be retained.

(1). Frequency analysis: If the responses are focused on a specific selection (more than 80%) or if a selection is not answered at all, delete.

(2). Coefficient of variation: In general, deletion can be considered when the coefficient of variation is < 0.25.

(3). High-low group comparison: The total scale scores were sorted from smallest to largest, and the score values corresponding to the 27th percentile and 73rd percentile were used as the upper limit for dividing the low group and the lower limit for the high group, respectively, to compare whether there was a difference between the scores of the low group and the high group on eachitem, and if there was no difference, they were deleted.

(4). Correlation coefficient method:

1) Internal item correlation coefficient method: The correlation coefficient r of each item and other items in its dimension is taken as the index. If *r* < 0.20 or *r* > 0.90, consider deleting it.
2) Item-dimension consistency method: In each dimension, the correlation coefficient r between each item and the score of the dimension after the removal of the item is taken as the index. If *r* < 0.20, it can be deleted.
3) Item-dimension correlation coefficient method: For each item, the correlation coefficient between the item and the dimension score after the removal of the item should be greater than the correlation coefficient between the item and the score of other dimensions; otherwise, the item should be considered for deletion.

(5). Factor analysis: Item deletion criteria: (a) the factor loading on the belonging factor is < 0.5; (b) the difference in factor loading on two or more factors is small (in this study, the difference in factor loading is not >0.05); (c) the belonging factor contains only one item (43).

(6). Cronbach's alpha coefficient method: If the Cronbach's alpha coefficient increases significantly after the removal of an item, it indicates that the item has the effect of reducing the internal consistency of this dimension, and can be deleted.

#### 2.3.2. Factor analysis

Exploratory factor analysis and confirmatory factor analysis assessed the construct validity of the scales. Exploratory factor analysis is typically used to distill a set of correlated data into a comprehensive factor structure, and confirmatory factor analysis is used to assess the fit of that factor structure. We randomly divided the collected data into two parts, one for exploratory factor analysis and one for confirmatory factor analysis. The items in the scale were grouped into several factors using principal component analysis and maximum variance rotation. It is generally accepted that factor analysis is meaningful only when the Kaiser-Meyer-Olkin (KMO) value is >0.7 and the Bartlett test is < 0.05 (44). The fit validity of the model was judged using the fit index, and the COMSIN manual proposed a strict criterion: χ^2^/df < 3, χ^2^ test results with *P* > 0.05, goodness of fit index >0.95 and root mean square error of approximation < 0.06 has good measurement properties (45, 46). The average variance extraction (AVE) and combination reliability were calculated on the basis of confirmatory factor analysis, and the square root of AVE of the dimension in question was generally considered to be greater than the correlation between the dimension and other dimensions, indicating a good discriminant validity.

#### 2.3.3. Reliability and validity

The performance evaluation of scales includes validity and reliability. Content validity index (CVI) is often used to measure content validity, including item level content validity index and scale level content validity index. In the process of expert inquiry by Delphi method, experts are asked to make judgments about the relevance of each item to the corresponding content dimension. Their judgments are divided into two parts, one that is considered relevant and one that is not, and the composition ratio of experts who consider the items relevant is calculated, namely, item-level CVI. It is generally considered that item-level CVI ≥ 0.78 represents better content validity at the itemlevel. In addition, the mean value of item-level CVI is often used to indicate scale-level CVI, and it is commonly thought that scale-level CVI ≥ 0.90 represents better content validity at the scale level (47, 48). Test-retest reliability and split-half reliability are commonly used to assess the reliability of the scale, it is generally expressed as the intraclass correlation coefficient (ICC) and the simple correlation coefficient (*r*), ICC or *r* >0.7 is generally considered a good confidence level. Cronbach's alpha coefficient was used to assess internal consistency and to test the degree of agreement between the scale and the internal items of each dimension.

#### 2.3.4. The assignment of scale scores

We eventually developed a “Systemic Sclerosis Health Literacy Scale” containing 6 dimensions and 30 items with a score of 30–150. The health literacy levels of SSc patients were classified into four levels according to the total scale scores of < 40%, 40–60%, 60–80% and more than 80%, namely low (30–60 score), limited (61–90 score), intermediate (91–120 score), adequate (121–150 score). A higher score on the scale means a higher level of health literacy.

## 3. Results

### 3.1. Characteristics of the SSc population

The study ultimately included 428 eligible study subjects. Among them, 364 were female, with a male to female ratio of 1:5.7, and the mean age of the patients was 51.57 ± 10.99, mainly middle-aged women. The majority of patients were from rural areas, predominantly farmers or otherwise working, and nearly a quarter of patients reported not working or being unable to work due to their disease. The overall education level of the patients was low, mainly the primary school education level, and only 17.9% of the patients had high school education or above. Most patients had a normal body mass index (BMI), some patients had symptoms of weight loss, and patients who were wasted or overweight accounted for about 33.7%. Nearly 90% of patients had limited systemic sclerosis, with a mean disease duration of 7.22 ± 6.89. More than 70% of patients had Raynaud's phenomenon, with common complications of ILD (34.6%) and PAH (28.0%) (Table 1).

**Table 1:** General demographic characteristics of SSc patients.

**Variables**		**Number (*N* = 428)**	**Percent (%)**
Age (years)		51.57 ± 10.99	–
Sex (female %)		364	85.0
Career	Farmer	99	23.1
	Public institutions and government officials	37	8.6
	Professionals	57	13.3
	Other	133	31.1
	None	102	23.9
Education level	Illiteracy	70	16.4
	Primary school	178	41.6
	Junior high school	103	24.1
	Senior high school/technical secondary school	38	8.9
	College/bachelor degree or above	39	9.0
BMI	< 18.4	61	14.3
	18.5–23.9	275	64.3
	24–27.9	83	19.4
	≥28	9	2.0
Course of disease	–	7.22 ± 6.89	–
SSc type, limited	–	384	89.7
Raynaud's phenomenon	–	301	70.3
Interstitial lung disease	–	148	34.6
Pulmonary artery hypertension	–	120	28.0

Data are expressed as mean ± SD or number (percentage).

### 3.2. Classical measurement theory screening items

#### 3.2.1. Frequency analysis method

The response rate of each item was 100%, and no item had a response rate of more than 80% on a certain option, and all items were retained.

#### 3.2.2. Coefficient of variation method

The range of score means for the 37 items was 2.06–4.14, the range of standard deviations was 0.611–1.265, and the range of coefficient of variation was 0.165–0.529. The coefficient of variation of 11 of the items was < 0.25, namely Q2.1, Q2.7, Q3.1, Q3.2, Q5.1, Q5.5, Q5.6, Q6.1, Q6.2, Q6.3, and Q6.4.

#### 3.2.3. High-low grouping comparison method

There were significant differences in the scores of all items between high and low groups (*P* < 0.001).

#### 3.2.4. Correlation coefficient method

1) Internal item correlation coefficient method: The correlation coefficient between item Q5.1 and the three items in the dimension is < 0.20, so consider deleting.

2) Item-dimension consistency method: The correlation coefficient between item Q6.1 and the scores of other items in the dimension is *r* = 0.143, so it is considered to be deleted.

3) Item-dimension correlation coefficient method: There are 6 items that meet the deletion criteria, that is, Q3.1, Q3.2, Q4.1, Q5.4, Q5.5, Q5.6.

#### 3.2.5. Factor analysis method

Items Q1.1, Q1.5, Q1.6, Q4.1 all have factor loadings in both dimensions and the difference is < 0.05.

#### 3.2.6. Cronbach's alpha coefficient method

The Cronbach's alpha coefficient for the social support dimension is 0.694, and after deleting Q6.1, the Cronbach's alpha coefficient rises to 0.763, and similarly, after deleting Q6.5, the Cronbach's alpha coefficient rises to 0.747. Therefore, deleting Q6.1 and Q6.5 is considered.

#### 3.2.7. Summary and analysis of item screening results

In the above 37 item screening analysis, item Q6.1 only satisfied four screening methods, so it was deleted. All other items meet the retention criteria (Table 2).

**Table 2:** Summary of items screening results.

**items**	**I**	**II**	**III**	**IV**			**V**	**VI**	**Number of standards achieved**	**Screening results**
				**i**	**ii**	**iii**				
Q1.1. You can find information about systemic sclerosis on the web	√	0.529	*P* < 0.001	0	0.813	0.746	0.599^*^	↓	7	Retain
Q1.2. You know the common signs and symptoms of systemic sclerosis	√	0.341	*P* < 0.001	0	0.757	0.660	0.739	↓	8	Retain
Q1.3. You can find information about complications of systemic sclerosis	√	0.370	*P* < 0.001	0	0.796	0.664	0.762	↓	8	Retain
Q1.4. You have actively searched for ways to improve your disease symptoms	√	0.264	*P* < 0.001	0	0.761	0.652	0.659	↓	8	Retain
Q1.5. You usually pay attention to information about health, such as food and nutrition, physical exercise, etc.	√	0.404	*P* < 0.001	0	0.771	0.701	0.527^*^	↓	7	Retain
Q1.6. You can find information on some common chronic diseases	√	0.433	*P* < 0.001	0	0.845	0.768	0.599^*^	↓	7	Retain
Q1.7. You can find information on mental health	√	0.484	*P* < 0.001	0	0.842	0.768	0.639	↓	8	Retain
Q2.1. You can understand the doctor's description of your condition	√	0.240^*^	*P* < 0.001	0	0.698	0.690	0.505	↓	7	Retain
Q2.2. You can understand the meaning of the medical written instructions	√	0.390	*P* < 0.001	0	0.880	0.789	0.661	↓	8	Retain
Q2.3. You can judge whether the lab index is normal or not according to the reference range on the lab report	√	0.419	*P* < 0.001	0	0.869	0.768	0.635	↓	8	Retain
Q2.4. You can read and understand drug instructions	√	0.441	*P* < 0.001	0	0.874	0.771	0.590	↓	8	Retain
Q2.5. You can understand the benefits and drawbacks of the medication prescribed by your doctor	√	0.363	*P* < 0.001	0	0.826	0.775	0.621	↓	8	Retain
Q2.6. You can read the signs in the hospital	√	0.293	*P* < 0.001	0	0.879	0.758	0.647	↓	8	Retain
Q2.7. You can understand your doctor's advice on your daily life	√	0.195^*^	*P* < 0.001	0	0.798	0.711	0.593	↓	7	Retain
Q3.1. You are able to clearly describe your symptoms and discomfort when talking to your doctor	√	0.227^*^	*P* < 0.001	0	0.703	0.750^*^	0.630	↓	6	Retain
Q3.2. You can understand most of what is said when you talk to the doctor	√	0.249^*^	*P* < 0.001	0	0.755	0.808^*^	0.664	↓	6	Retain
Q3.3. When in doubt about medical advice, you proactively ask your doctor	√	0.281	*P* < 0.001	0	0.842	0.755	0.758	↓	8	Retain
Q3.4. You will check with your doctor to make sure that you understand the medical advice correctly	√	0.321	*P* < 0.001	0	0.800	0.675	0.762	↓	8	Retain
Q3.5. You will discuss treatment options with your doctor	√	0.415	*P* < 0.001	0	0.756	0.669	0.697	↓	8	Retain
Q3.6. You will ask your doctor for the tests or treatments you want	√	0.498	*P* < 0.001	0	0.758	0.747	0.685	↓	8	Retain
Q3.7. You will discuss health issues with people other than your doctor	√	0.391	*P* < 0.001	0	0.723	0.67	0.527	↓	8	Retain
Q4.1. You can judge whether what the doctor says fits your condition	√	0.378	*P* < 0.001	0	0.788	0.805^*^	0.589^*^	↓	6	Retain
Q4.2. You can determine if the information you receive about systemic sclerosis is correct	√	0.464	*P* < 0.001	0	0.894	0.791	0.768	↓	8	Retain
Q4.3. You can judge the usefulness of the information you receive about systemic sclerosis	√	0.449	*P* < 0.001	0	0.887	0.786	0.747	↓	8	Retain
Q4.4. You can make medical decisions based on the information collected about your disease	√	0.357	*P* < 0.001	0	0.840	0.736	0.686	↓	8	Retain
Q4.5. You will change doctors to expect a different opinion	√	0.292	*P* < 0.001	0	0.629	0.557	0.600	↓	8	Retain
Q5.1. You will take your medication in strict accordance with your doctor's instructions or the drug instructions	√	0.211^*^	*P* < 0.001	3^*^	0.473	0.24	0.866	↓	6	Retain
Q5.2. You will not reduce or stop your medication without consulting your doctor	√	0.269	*P* < 0.001	2	0.576	0.255	0.862	↓	8	Retain
Q5.3. You will come to the hospital for regular review	√	0.303	*P* < 0.001	0	0.483	0.218	0.605	↓	8	Retain
Q5.4. You can know exactly how your disease is developing	√	0.263	*P* < 0.001	1	0.468	0.665^*^	0.576	↓	7	Retain
Q5.5. You will engage in behaviors that will improve your health	√	0.200^*^	*P* < 0.001	2	0.454	0.466^*^	0.608	↓	6	Retain
Q5.6. You can take a positive approach to coping with the stress of the disease	√	0.208^*^	*P* < 0.001	0	0.367	0.471^*^	0.609	↓	6	Retain
Q6.1. Have a family member or friend with you at your doctor's appointment	√	0.220^*^	*P* < 0.001	2^*^	0.143^*^	−0.110	0.522	↑^*^	4	Delete
Q6.2. For those who do not understand the information, you will have a family member or friend or medical staff to help you understand	√	0.165^*^	*P* < 0.001	0	0.626	0.254	0.812	↓	7	Retain
Q6.3. When you feel uncomfortable, you are surrounded by people who understand what you are going through	√	0.190^*^	*P* < 0.001	0	0.639	0.339	0.799	↓	7	Retain
Q6.4. If you need help, you have reliable people around you	√	0.215^*^	*P* < 0.001	0	0.630	0.302	0.865	↓	7	Retain
Q6.5. You understand and apply for medical coverage	√	0.268	*P* < 0.001	0	0.242	0.384^*^	0.532	↑^*^	6	Retain

I, Frequency analysis method; II, Coefficient of variation method; III, High-low grouping comparison method; IV(i), Internal item correlation coefficient method; IV(ii), Item-dimension consistency method; IV(iii), Item-dimension correlation coefficient method; V. Factor analysis method; VI, Cronbach's alpha coefficient method.

^*^Meet the deletion criteria.

“√” means meeting the inclusion requirements of frequency analysis; “↑” indicates an increase in Cronbach's alpha coefficient after excluding the item; “↓” indicates a decrease in Cronbach's alpha coefficient after excluding this item.

### 3.3. Factor analysis

The 214 collected data were subjected to exploratory factor analysis with a KMO value of 0.949 and Bartlett's spherical test *P* < 0.001, and the data were suitable for factor analysis. After removing item Q6.1, six common factors are extracted based on the eigenvalues >1, and the cumulative contribution rate of variance is 72.680%. Among them, items Q1.6, Q3.7, and Q4.1 are distributed in two dimensions and the difference of factor loadings is < 0.05, so they are deleted. The factor loading of item Q6.5 is < 0.5, so it is deleted (Table 3).

**Table 3:** Each factor rotated component matrix.

	**Factor 1**	**Factor 2**	**Factor 3**	**Factor 4**	**Factor 5**	**Factor 6**	**Extraction**
Q1.1			0.621				0.758
Q1.2			0.739				0.754
Q1.3			0.765				0.790
Q1.4			0.658				0.689
Q1.5			0.548				0.687
Q1.7		0.597					0.798
Q2.1	0.515						0.659
Q2.2	0.677						0.788
Q2.3	0.653						0.759
Q2.4	0.613						0.763
Q2.5	0.642						0.741
Q2.6	0.665						0.765
Q2.7	0.608						0.740
Q3.1	0.639						0.754
Q3.2	0.673						0.780
Q3.3	0.760						0.776
Q3.4	0.757						0.713
Q3.5	0.696						0.657
Q3.6		0.669					0.732
Q4.2		0.749					0.839
Q4.3		0.725					0.842
Q4.4		0.680					0.769
Q4.5		0.614					0.535
Q5.1						0.879	0.826
Q5.2						0.870	0.836
Q5.3						0.564	0.596
Q5.4					0.584		0.702
Q5.5					0.650		0.611
Q5.6					0.599		0.560
Q6.2				0.812			0.715
Q6.3				0.840			0.781
Q6.4				0.885			0.829

Factor 1, clinic ability; Factor 2, judgment/evaluation information ability; Factor 3, access to information ability; Factor 4, social support; Factor 5, treatment compliance; Factor 6, application information ability.

Finally, 32 items with 6 dimensions were retained. Dimension 1 includes 12 items to evaluate patients' ability to understand and communicate information, that is, patients' ability to attend the clinic; dimension 2 includes 6 items to evaluate patients' ability to judge / assess information; dimension 3 includes 5 items to evaluate patients' ability to obtain information; dimension 4 includes 3 items to evaluate social support; dimension 5 includes 3 items to evaluate patients' ability to apply information; Dimension 6 includes 3 items to evaluate the regularity of patients' medication and review, that is, patients' treatment compliance.

**Figure 2:**
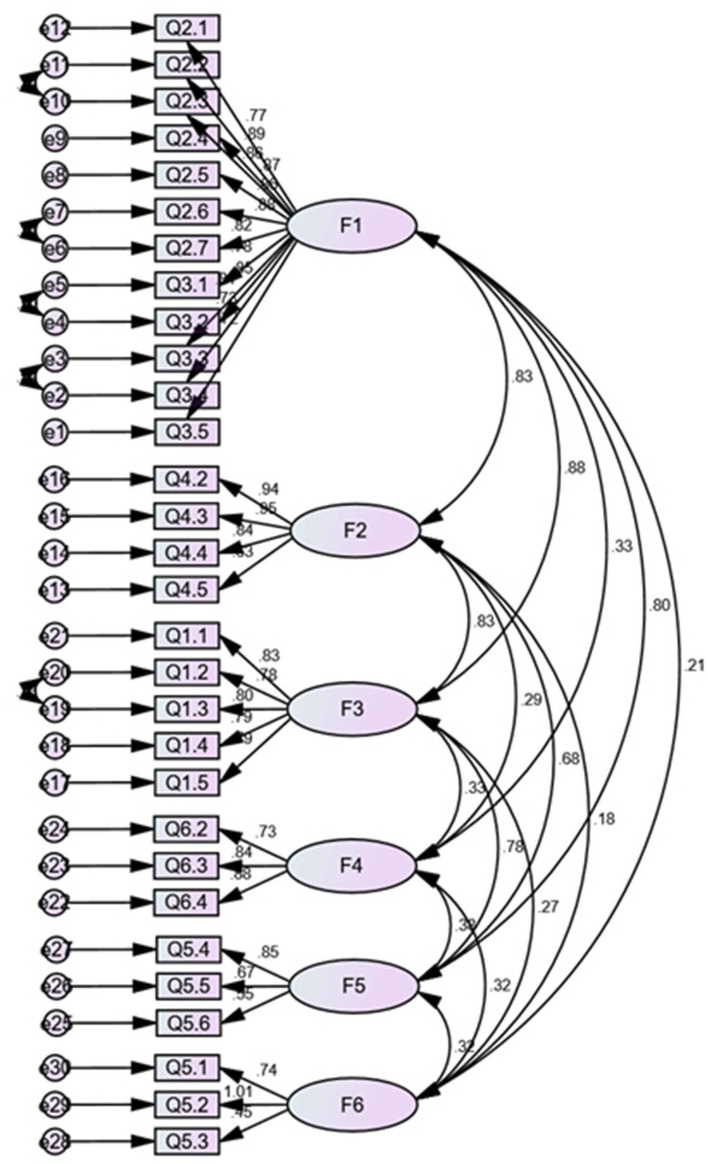
Structural validity of confirmatory factor analysis.

The confirmatory factor analysis was performed on the remaining 214 samples to explore the construct validity of the scale (Figure 2). The results of the dimensions that Q1.7 and Q3.6 belonged to in the exploratory factor analysis were different from those initially classified, and we fitted the model to each of the four cases, including retaining Q1.7 and Q3.6, deleting one of them, and deleting both. The results showed that deleting Q1.7 and Q3.6 had the best fit validity. After consulting with experts, we decided to delete these two items and keep the remaining ones. The results of the final scale fit showed that X^2^/df = 1.798 < 3, RMSEA = 0.061, GFI = 0.945, IFI = 0.945, TLI = 0.937, which are close to COSMIN criteria, implying that the overall fit validity of the scale did not meet the criteria of goodness of fit. However, according to the COMSIN manual's comprehensive consideration of measurement standards, this study result is completely acceptable.

On the basis of construct validity, the aggregate validity of the scale was evaluated according to average variance extraction (AVE) and combination reliability (Table 4). The results showed that the square root of AVE for dimension 1 was smaller than the maximum value of the absolute value of its inter-factor correlation coefficient of 0.879, implying slightly poorer discriminant validity, but all other dimensions showed better discriminant validity, and we considered the overall convergent validity and discriminant validity of the scale to be up to standard.

**Table 4:** Discriminant validity for SSc health literacy scale.

	**Factor 1**	**Factor 2**	**Factor 3**	**Factor 4**	**Factor 5**	**Factor 6**
Factor 1	0.675	0.723	0.637	0.671	0.496	0.582
Factor 2	0.834					
Factor 3	0.879	0.828				
Factor 4	0.329	0.287	0.334			
Factor 5	0.800	0.683	0.778	0.383		
Factor 6	0.208	0.184	0.270	0.323	0.316	
AVE square root	0.821	0.850	0.798	0.820	0.704	0.762

Factor 1, clinic ability; Factor 2, judgment/evaluation information ability; Factor 3, access to information ability; Factor 4, social support; Factor 5, treatment compliance; Factor 6, application information ability.

### 3.4. Performance evaluation of the scale

#### 3.4.1. Validity evaluation

Content validity: The item-level CVI ranges from 0.875 to 1.000, and the scale-level CVI is 0.983. The results all meet the criteria, indicating that the overall content validity of the scale is good.

#### 3.4.2. Reliability evaluation

Test-retest reliability: A sample of 50 people was taken for a second survey within 2 weeks after the first survey, and 49 valid questionnaires were returned. The intraclass correlation coefficient was calculated for each item of the two measurements and the total scale, and the results showed that the intraclass correlation coefficient ranged from 0.712 to 0.851, and the total scale intraclass correlation coefficient was 0.898 (*p* < 0.05), which indicates that the stability of the scale is good.

Split-half reliability: The items were divided into two equal parts, namely even-numbered items and odd-numbered items, and the correlation coefficient between the two parts was calculated (*r* = 0.953, *P* < 0.001).

Internal consistency: The internal consistency of the scale is often assessed by the Cronbach's alpha coefficient of each dimension, and a Cronbach's alpha coefficient of more than 0.7 for each dimension indicates good internal consistency of the scale (Table 5).

**Table 5:** Cronbach's alpha coefficient for SSc health literacy scale.

**Dimensions**	**Cronbach's alpha coefficient**	**Eigenvalues**	**Cumulative contribution rate (%)**
Clinic ability	0.961	18.154	21.371
Judgment/evaluation information ability	0.909	2.616	37.378
Access to information ability	0.903	1.704	51.635
Social support	0.715	1.378	59.311
Treatment compliance	0.855	1.223	66.749
Application information ability	0.729	1.090	72.680

### 3.5. Health literacy level of SSc patients

In this study, the percentage of patients with adequate health literacy level was 14.49%, which is close to the health literacy level of the general population (14.18%) reported in 2017 in China (49). In our collection, SSc patients had extremely low levels of health literacy in terms of finding health information (10.3%) and assessing health information dimensions (8.0%); more than half (52.8%) showed good treatment compliance, but this was not enough; and nearly half (48.1%) reported being able to use the information they already had to help them slow the progression of their disease. Only 26.2% of the patients indicated that they had sufficient medical treatment ability and could make use of medical service resources very effectively, and 29.4% of the patients had adequate social support.

Additionally, we assessed the health literacy levels of patients of different ages and education levels (Table 6). It can be seen that with the increase of age, the scale score is gradually declining, except for dimension 4 “social support,” the scores of other dimensions show a downward trend. Spearman correlation coefficient showed that age was negatively correlated with health literacy level (*r*_s_ = −0.321, *p* < 0.05). But, as we suspected, health literacy scores increased with education, and this phenomenon also showed up in all dimensions except for dimension 6, “treatment compliance.” Spearman correlation coefficient showed that education level was positively correlated with health literacy level (*r*_s_ = 0.654, *p* < 0.05).

**Table 6:** Health literacy scores of patients of different ages and education levels.

**Variables**		**Number (*N* = 428)**	**Score on “Systemic Sclerosis Health Literacy Scale”**						
			**Dimension 1 (total: 60)**	**Dimension 2 (total: 20)**	**Dimension 3 (total: 25)**	**Dimension 4 (total: 15)**	**Dimension 5 (total: 15)**	**Dimension 6 (total: 15)**	**Total score: (150)**
	< 40	64	45.22 ± 8.38	12.88 ± 3.30	16.63 ± 3.88	10.72 ± 1.71	11.50 ± 2.00	11.87 ± 1.83	108.81 ± 16.72
	40–49	77	40.95 ± 10.77	11.08 ± 3.44	14.43 ± 4.49	10.11 ± 2.17	11.27 ± 1.98	11.68 ± 2.95	99.51 ± 22.05
Age (years)	50–59	202	38.13 ± 9.26	10.05 ± 3.36	13.33 ± 3.98	10.30 ± 1.60	11.10 ± 1.90	11.48 ± 2.37	94.38 ± 17.83
	60–69	71	38.03 ± 8.85	9.83 ± 3.37	12.71 ± 4.28	10.40 ± 1.70	11.09 ± 2.01	11.31 ± 2.69	93.37 ± 18.27
	>70	14	26.22 ± 13.05	7.89 ± 3.66	9.56 ± 4.90	10.67 ± 1.41	9.44 ± 2.56	10.89 ± 2.03	74.67 ± 22.88
	Illiteracy	70	27.88 ± 8.06	7.13 ± 2.28	9.90 ± 3.67	9.97 ± 1.85	9.63 ± 2.48	10.88 ± 2.49	75.38 ± 16.06
	Primary school	178	37.58 ± 8.63	9.80 ± 3.04	12.91 ± 3.70	10.20 ± 1.59	11.01 ± 1.67	11.48 ± 2.56	92.98 ± 16.57
Education level	Junior high school	103	43.56 ± 6.75	12.04 ± 2.76	15.10 ± 3.71	10.22 ± 1.74	11.56 ± 1.55	12.02 ± 2.45	104.50 ± 13.85
	Senior high school/technical secondary school	38	47.74 ± 6.13	12.79 ± 3.01	17.05 ± 3.19	11.11 ± 1.37	12.26 ± 1.52	11.79 ± 1.78	112.74 ± 12.60
	College/bachelor degree or above	39	49.89 ± 3.90	14.68 ± 2.34	18.84 ± 2.32	11.53 ± 1.84	12.42 ± 1.71	11.47 ± 2.17	118.84 ± 9.05

Dimension 1, clinic ability; Dimension 2, judgment/evaluation information ability; Dimension 3, access to information ability; Dimension 4, social support; Dimension 5, treatment compliance; Dimension 6, application information ability.

## 4. Discussion

This study was the first to develop a health literacy scale based on factor analysis and reliability evaluation to assess the health literacy level of SSc patients. The SSc health literacy scale contains six dimensions with 30 items. The results showed that the scale has good validity and reliability and may become a valid assessment tool.

In the original design of the scale, understanding and communicating health information are two separate dimensions, but in our results, the comprehension and communication ability of SSc patients influence each other greatly. This result may be related to the sample size, as a larger sample size can be used to assess more information and compensate for smaller differences. The larger the sample size, the more it reflects the patient's true ability to understand and communicate information (50). We define the ability to understand and communicate information together as clinic ability.

Before the scale was developed, we referred to existing scales and classified patients' medication-taking and regular review behaviors as applied competencies (38, 51). In the results of our study, although some patients do not have a clear understanding of their condition and do not take additional measures to improve their health, they have high drug compliance and are subject to regular reexaminations. This is one factor for which some researchers have postulated that no association was shown between health literacy and medication adherence (52). Therefore, we define these items as treatment compliance and define patients' application ability to make some behaviors conducive to improving the disease according to the progress of the disease.

Most importantly, we have focused here on the social support of the patient. The help of medical staff and the support of family members all contribute to the improvement of the patient's disease and influence the impact of the patient's health literacy level on clinical outcomes (53). At the same time, some studies suggest that people with systemic sclerosis may benefit from the social support of intimate relationships (54).

Currently, the “China Health Literacy Monitoring Questionnaire” is widely used to assess the health literacy level of the Chinese population, including those with chronic diseases (55, 56). The scale mainly assesses the level of health knowledge, disease prevention awareness, and emergency skills of the study population and is not specific to diseases (49). Moreover, the “health literacy scale for chronic patients” has been frequently used in research studies and includes four dimensions: access to information, communication of interactive information, willingness to improve health, and financial support (57). The scale developed in this study also assesses patients' ability to understand and evaluate information based on these scales. The majority of patients in our study results reported having applied for medical services to reduce the financial burden, so we did not factor in economics. Our emphasis on social support was more focused on the whole range of social concerns about the patient's consultation process, outcomes, and psychological aspects.

We also assessed the health literacy level of SSc patients, which was basically close to that of the general Chinese population. Among them, patients were the least able to assess information and had difficulty discriminating between the health information obtained, which is coherent with the results of other population studies on health literacy (58). Our results show that few patients are proactive in accessing health information and have less health information, but can use their limited health knowledge to manage their disease. SSc patients show the same characteristics in terms of access to electronic information (31). As a result, we should focus on the level of health knowledge of patients and increase health promotion and education, so that patients have more understanding of health, so as to make use of more health information.

In this study, few patients were able to make adequate use of medical information, which may result in patients repeatedly using medical resources or even appearing to be unable to use them correctly. Treatment adherence is crucial in the long-term treatment of SSc, but our findings show that only half of the patients have good adherence, while others experience poor medication adherence and irregular reviews. This result is in line with the results of a study on the knowledge of medication use in patients with chronic diseases (59).

The study also found a correlation between age, education level and health literacy among SSc patients. This finding is consistent with the results of other studies (60, 61). However, when analyzing each dimension specifically, it was found that the dimension “social support” did not decrease with age, where the younger group indicated that they had access to more policy information, more medical content, and could actively obtain more social support. However, with the increase of age, patients over 40 years old will receive different attention and social support, instead, elderly people will get more care and help. In the analysis of education levels and health literacy, it was found that patients with higher education levels had poorer treatment compliance, which may be due to the fact that patients with higher education levels undertake more social work, leading to delayed medical treatment. It is also possible that this group does not have enough health awareness and will make wrong decisions based on their own ideas.

The current study developed the SSc health literacy scale and assessed the health literacy level of this group. Although the final evaluation of the validity and reliability of the scale is good, the study also has some limitations. Firstly, the cross-cultural applicability of the “Systemic Sclerosis Health Literacy Scale” is unclear because this scale was developed and validated based on Chinese populations and Chinese medical settings, and most of the current papers are written in English, the ease of finding accurate medical information differs for those who cannot read English and those who can read English. Several studies have shown that English literacy is independently associated with seeking health information, that people with lower English proficiency also have lower utilization of health information, and that respondents who use Chinese have higher rates of limited health literacy than those who speak English (62, 63). Next, the subjects of this study were mainly from the First Affiliated Hospital of Anhui Medical University and the First Affiliated Hospital of China Medical University, and most of the patients had a low literacy level and an average economic level, which may have created a selection bias.

## 5. Conclusion

In short, this study focused for the first time on the health literacy level of SSc patients and developed the SSc Health Literacy Scale with 6 dimensions and 30 items. The scale has high reliability and validity, and the items are relatively simple and the time is short. The scale can be developed as a health literacy assessment tool for SSc patients and identify key issues such as patients' ability to see a doctor.

## Data availability statement

The raw data supporting the conclusions of this article will be made available by the authors, without undue reservation.

## Ethics statement

The studies involving human participants were reviewed and approved by Biomedical Ethics Committee of Anhui Medical University (number 20210649). The patients/participants provided their written informed consent to participate in this study.

## Author contributions

MZ, C-CL, S-YC, X-HT, and LL completed the data collection and organization. MZ, X-LC, C-WX, and JW analyzed the data, and the first draft of the manuscript was completed jointly by MZ, C-CL, S-YC, and JW. All authors contributed to the study design and process, approved, and ratified the final manuscript.
